# Supplementary material for: Characterizing the Interplay of Rubisco and Nitrogenase Enzymes in Anaerobic-Photoheterotrophically Grown Rhodopseudomonas palustris CGA009 through a Genome-Scale Metabolic and Expression Model
Source: Microbiol Spectr. 2022 Jun 22;10(4):e01463-22. doi: 10.1128/spectrum.01463-22 (PMC9431616; doi:10.1128/spectrum.01463-22)
Supplement: Supplemental file 1 — Supplemental material. Download spectrum.01463-22-s0007.pdf, PDF file, 0.2 MB [file spectrum.01463-22-s0007.pdf]

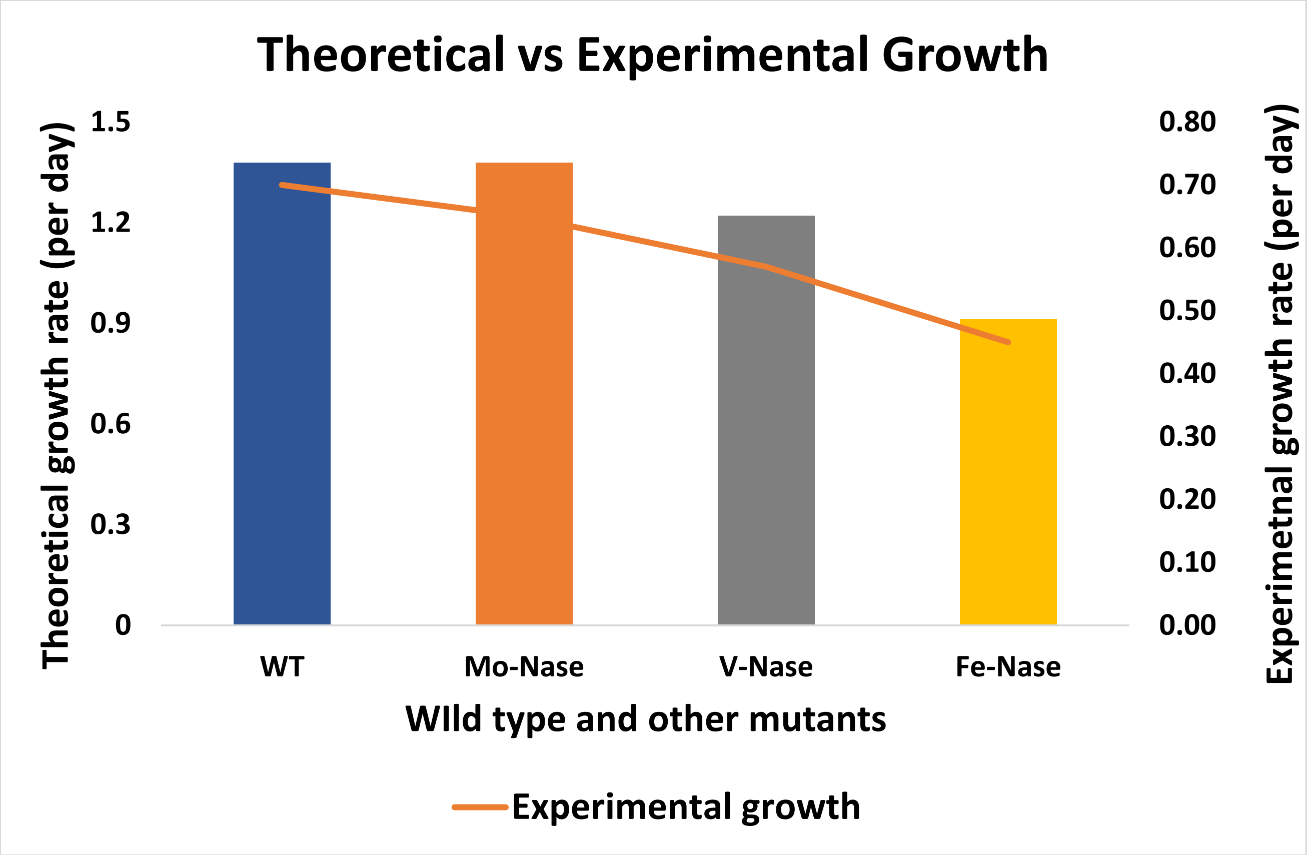

FIG S1 Comparison between theoretical maximum growth found from the ME-modeling framework and the experimental work from Luxem et al. 2020 when succinate was used as substrate.

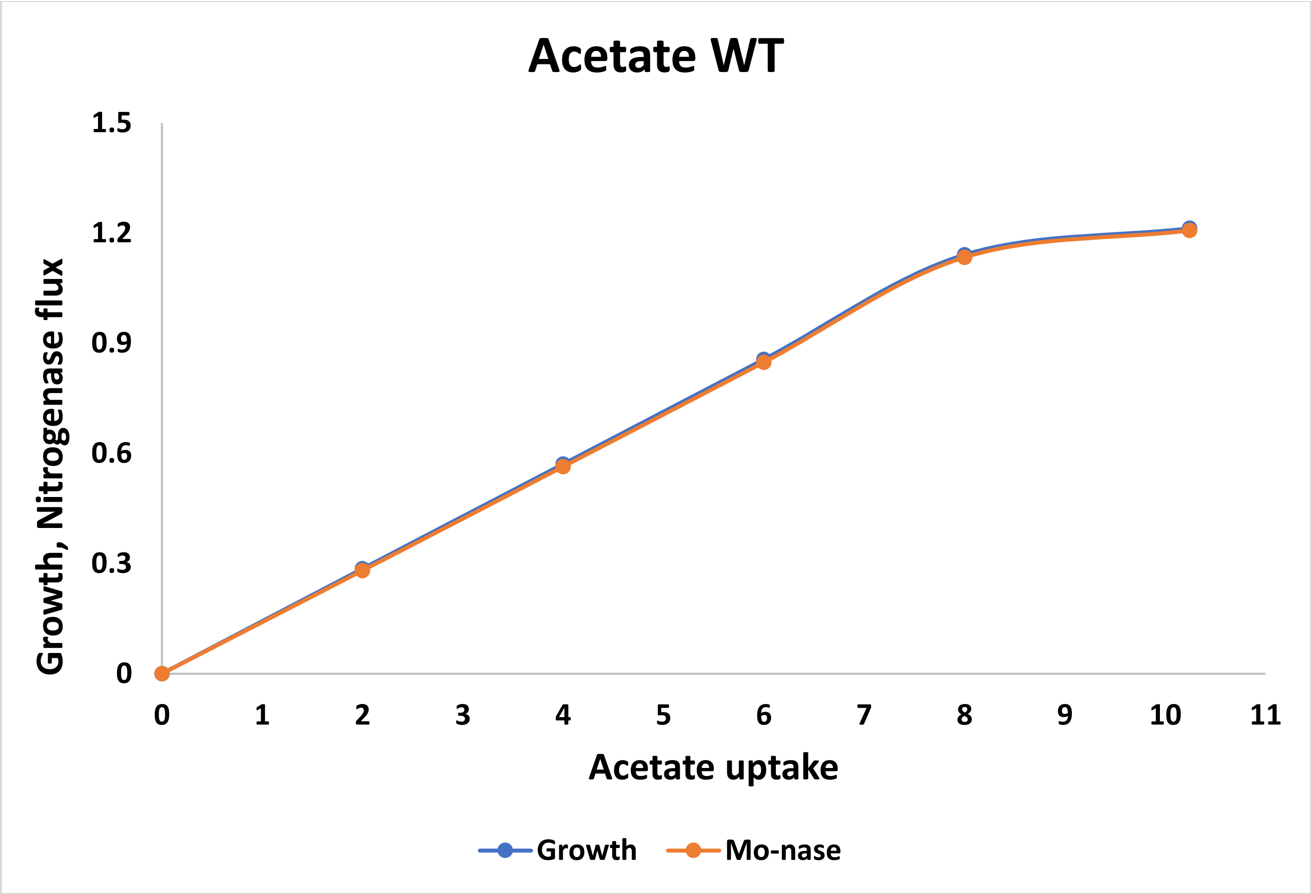

FIG S2 (a) For acetate uptake, growth rate and nitrogen fixation rate for WT *R. palustris*. For each of the case, growth rate and nitrogen fixation closely follow each other.

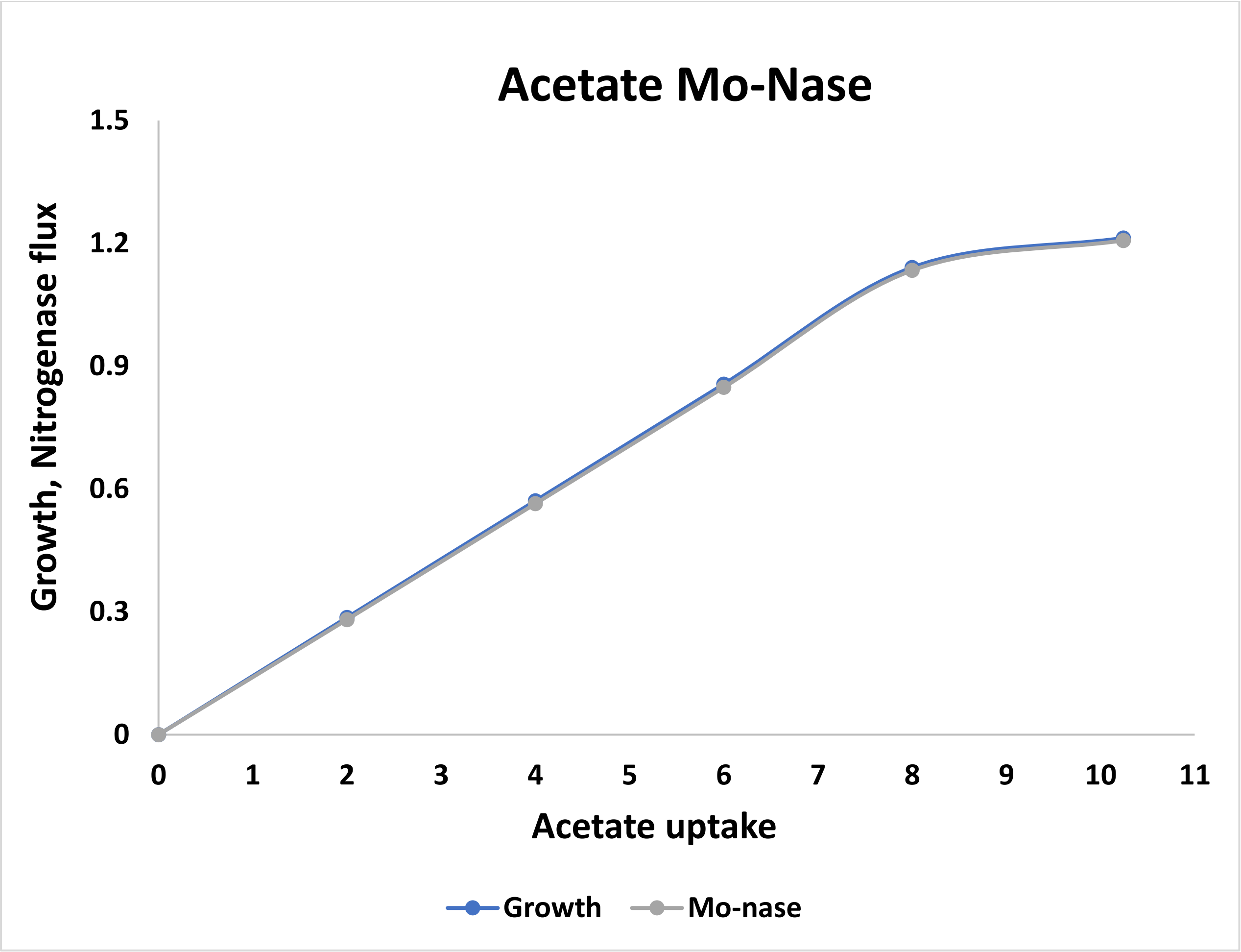

FIG S2 (b) For acetate uptake, growth rate and nitrogen fixation rate for Mo-Only mutant. For each of the case, growth rate and nitrogen fixation closely follow each other.

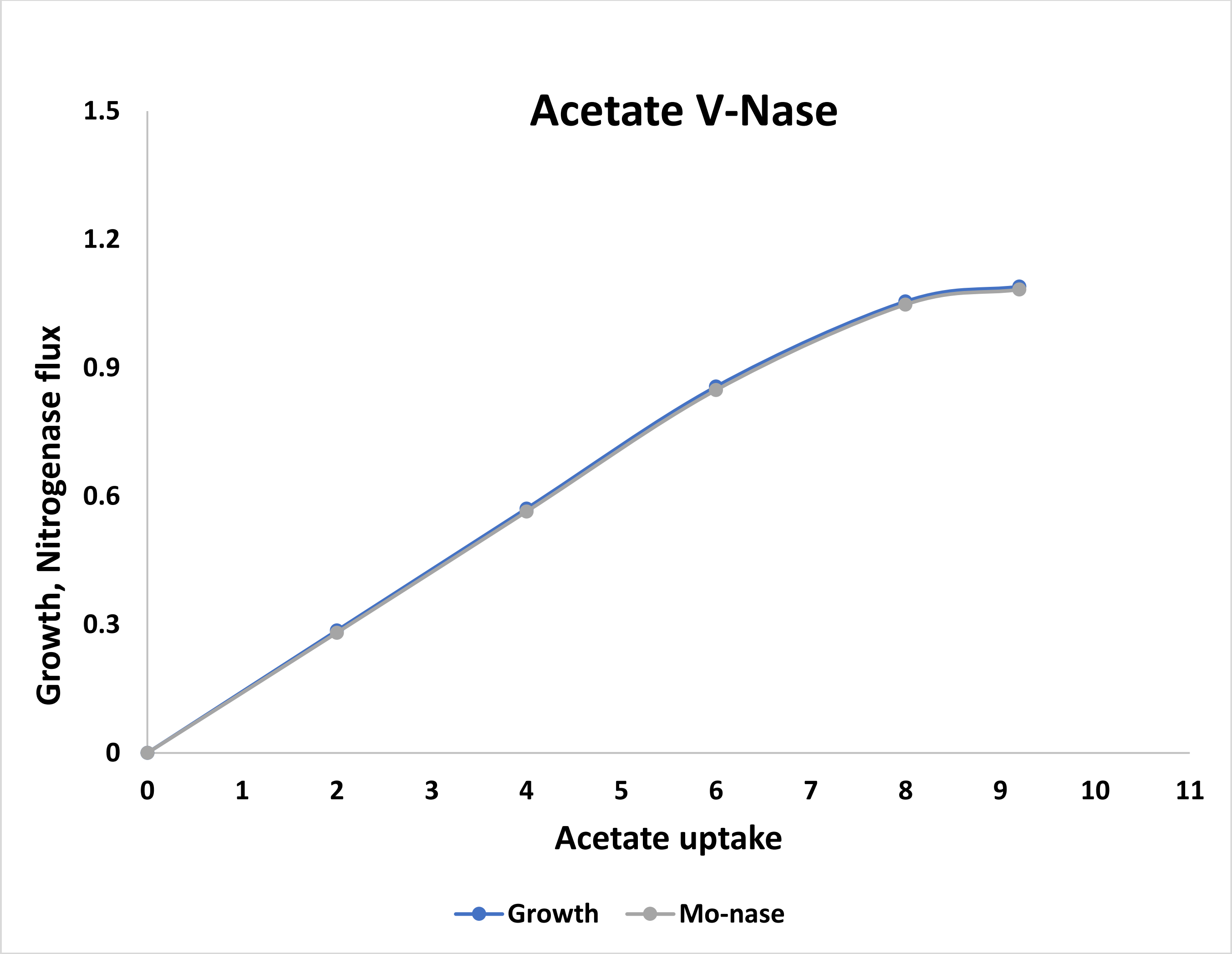

FIG S2 (c) For acetate uptake, growth rate and nitrogen fixation rate for V-Only mutant. For each of the case, growth rate and nitrogen fixation closely follow each other.

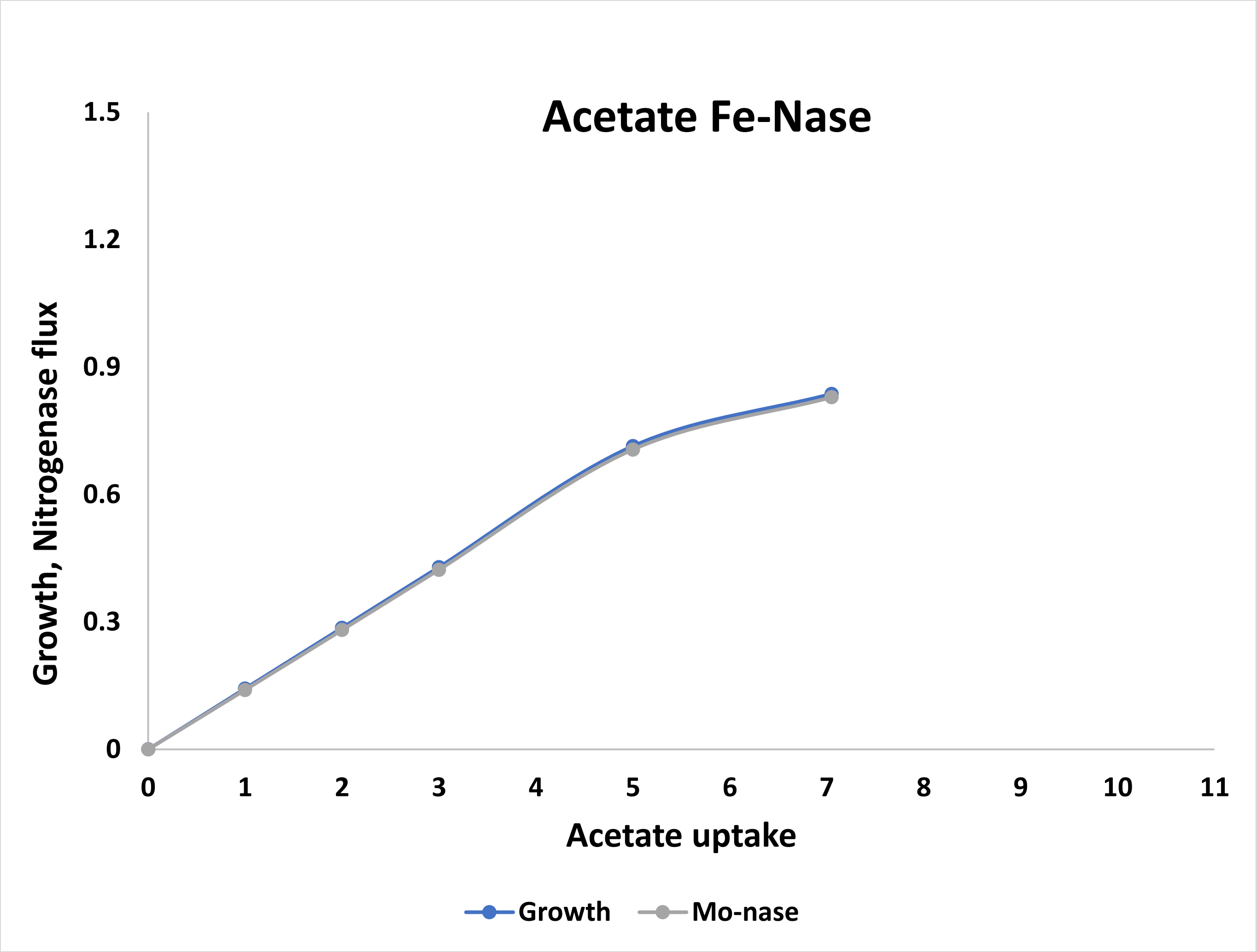

FIG S2 (d) For acetate uptake, growth rate and nitrogen fixation rate for Fe-Only mutant. For each of the case, growth rate and nitrogen fixation closely follow each other.

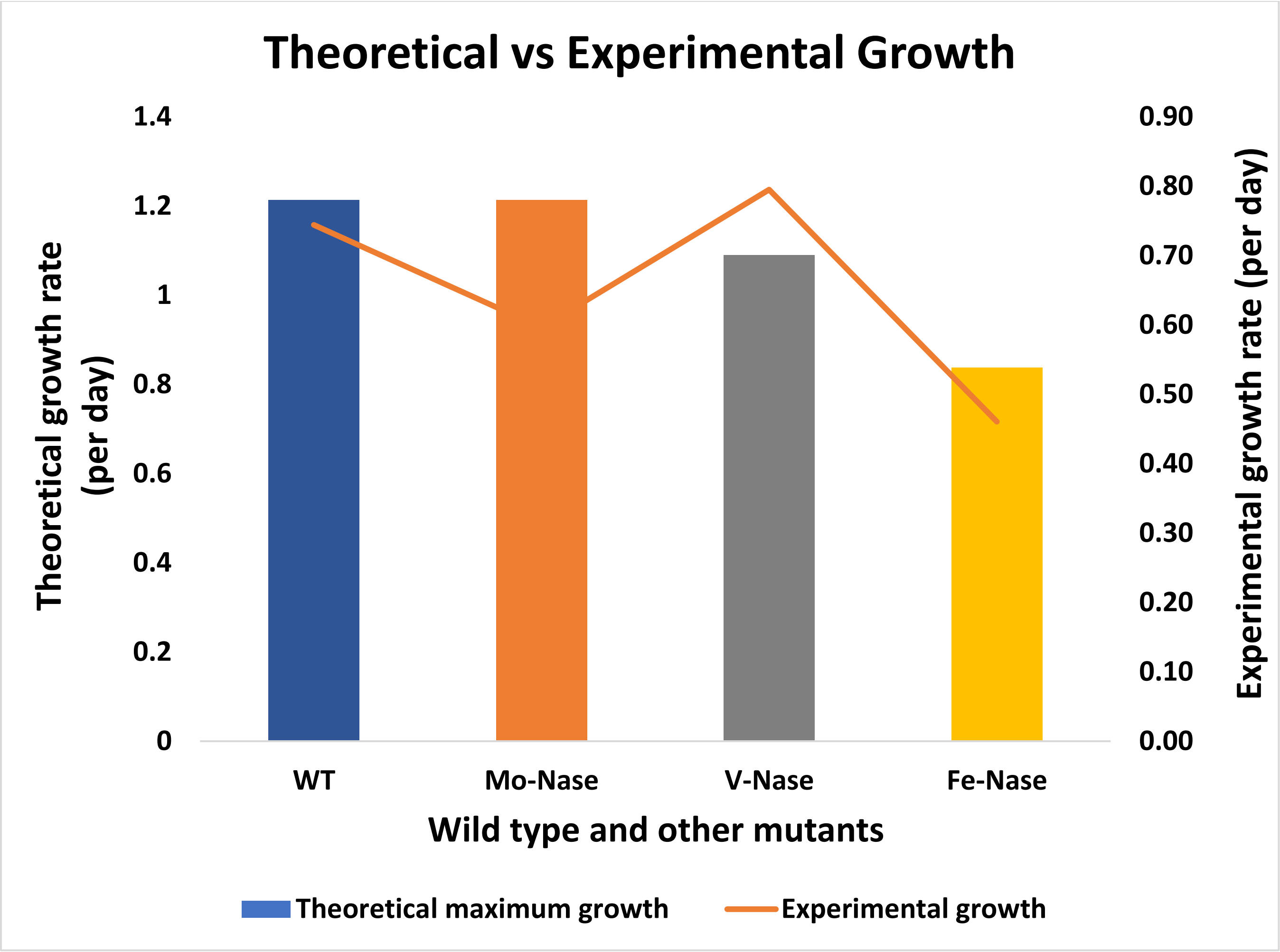

(e)

FIG S2 (e) For acetate uptake, comparison between theoretical maximum growth found from the ME-modeling framework and the experimental work from Luxem et al. 2020 when acetate was used as substrate.

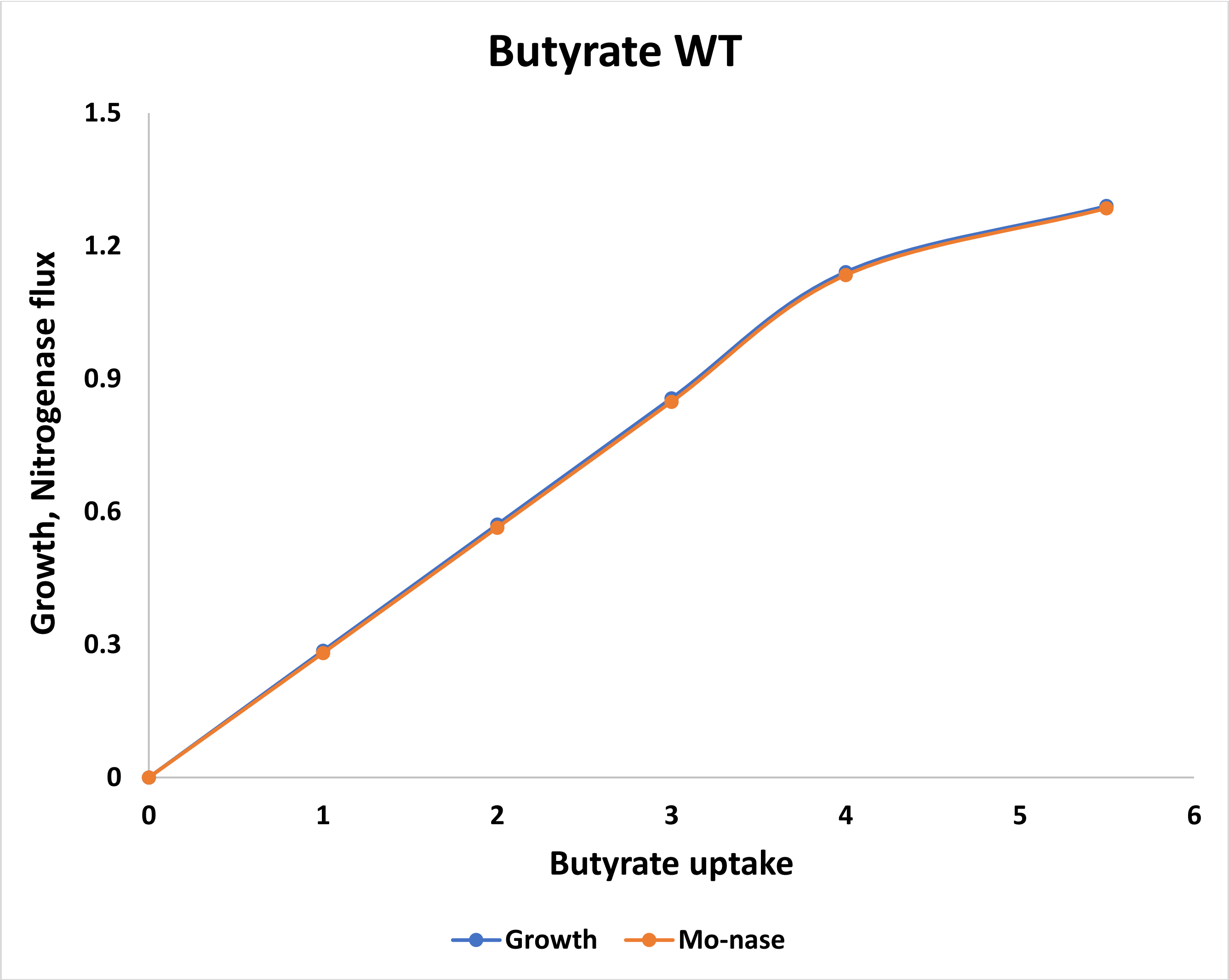

FIG S3 (a) For butyrate uptake growth rate and nitrogen fixation rate for WT *R. palustris*. For each of the case, growth rate and nitrogen fixation closely follow each other.

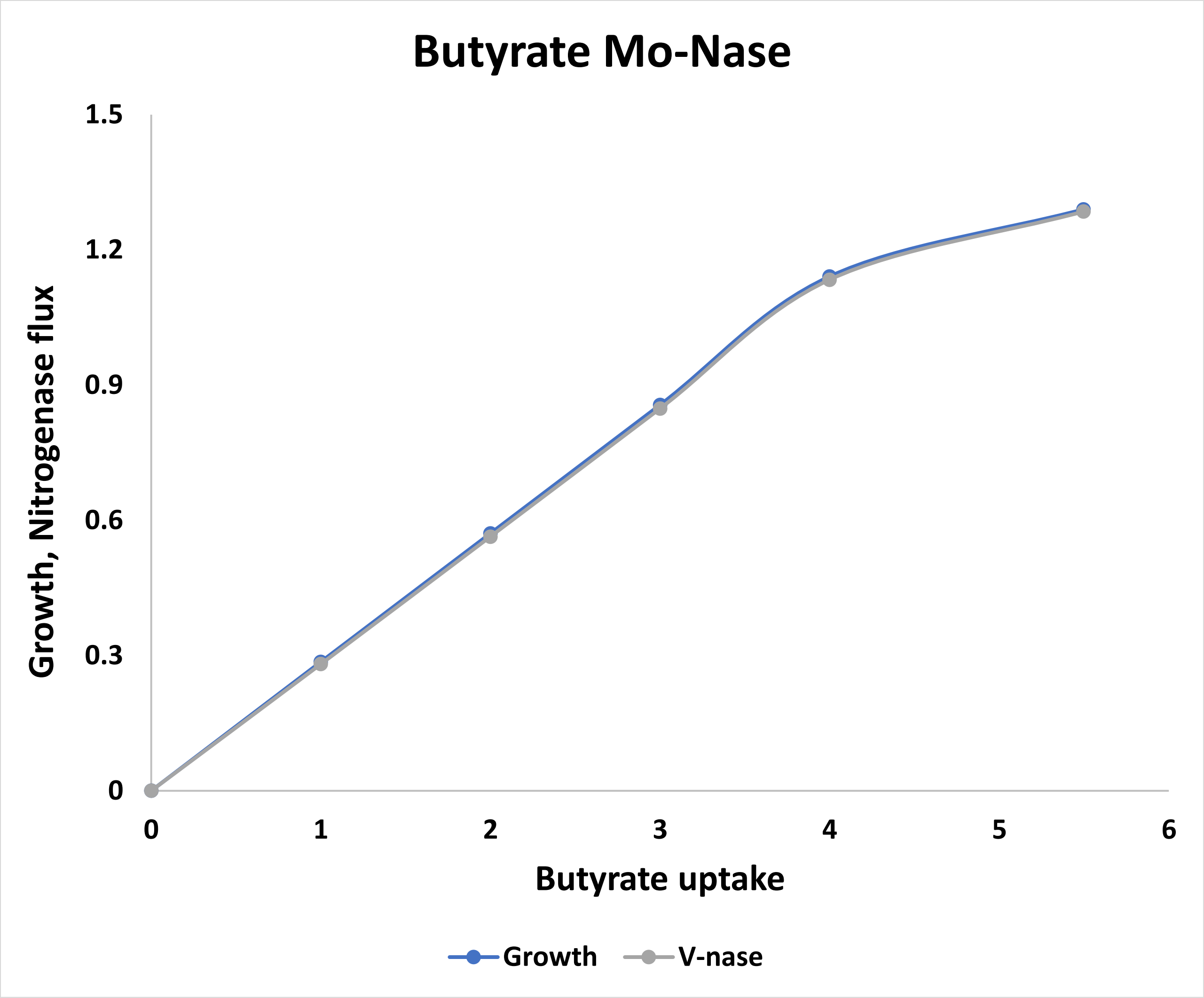

FIG S3 (b) For butyrate uptake growth rate and nitrogen fixation rate for Mo-only mutant. . For each of the case, growth rate and nitrogen fixation closely follow each other.

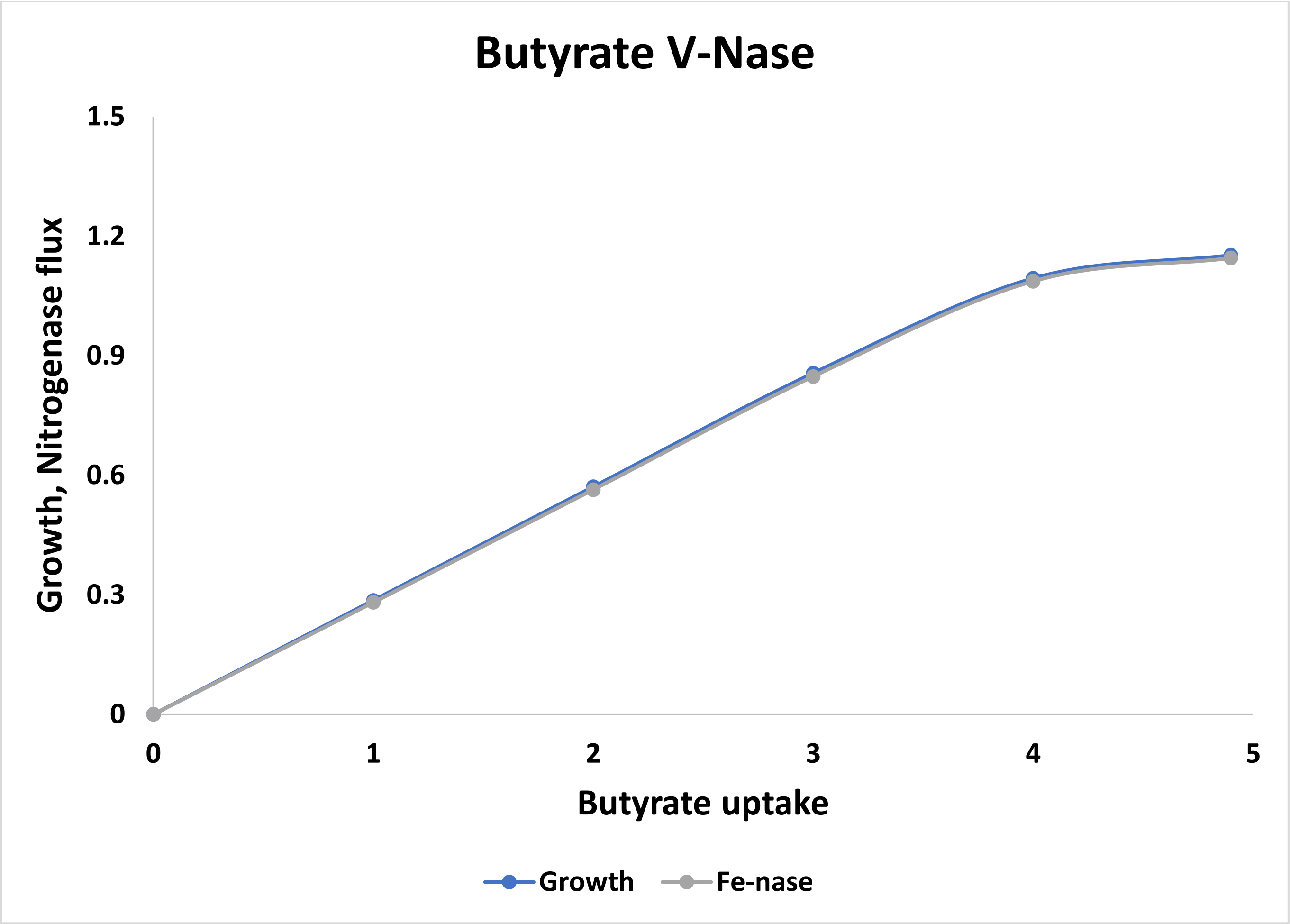

FIG S3 (c) For butyrate uptake growth rate and nitrogen fixation rate for V-only mutant. . For each of the case, growth rate and nitrogen fixation closely follow each other.

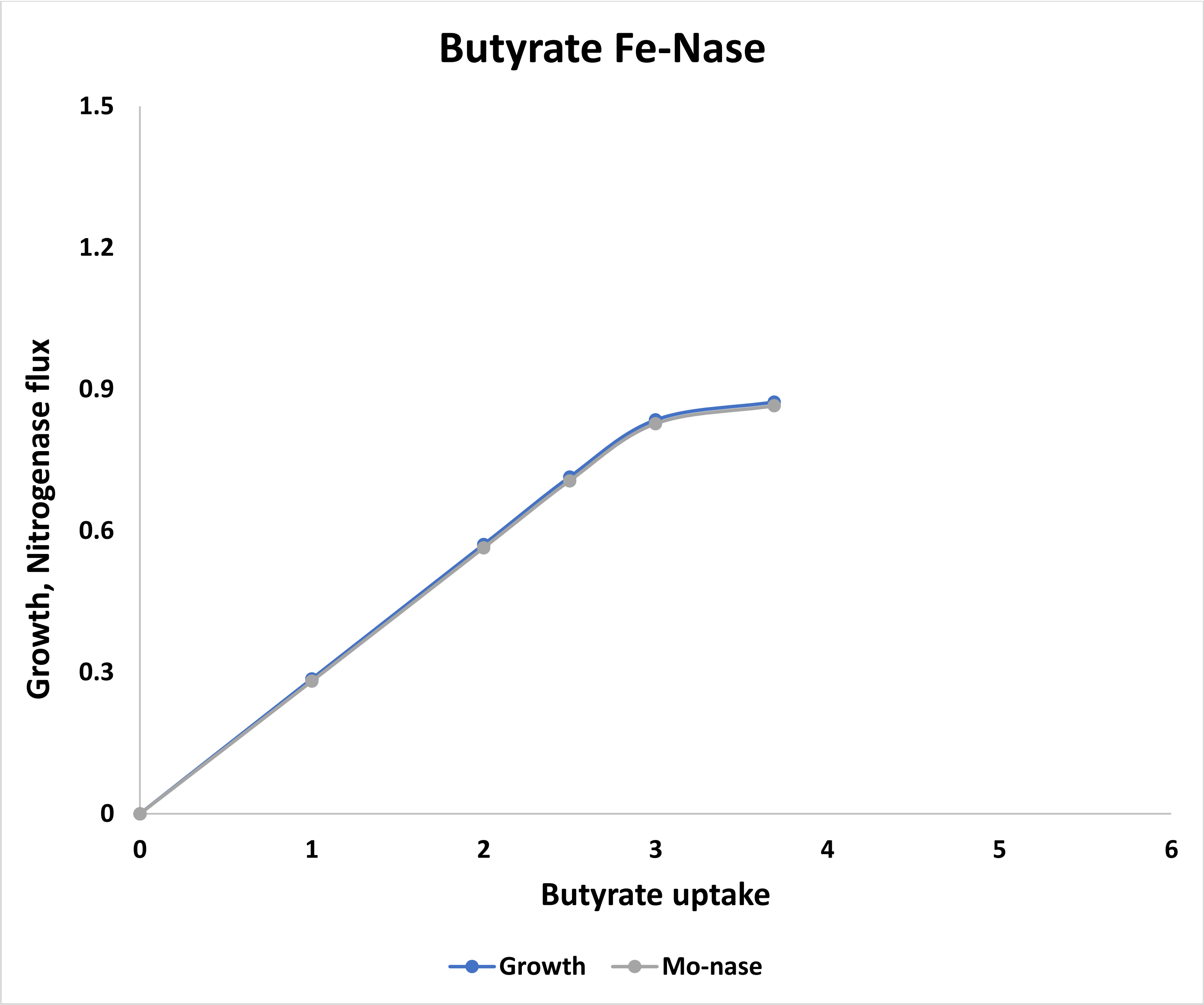

FIG S3 (d) For butyrate uptake growth rate and nitrogen fixation rate for Fe-only mutant. For each of the case, growth rate and nitrogen fixation closely follow each other.

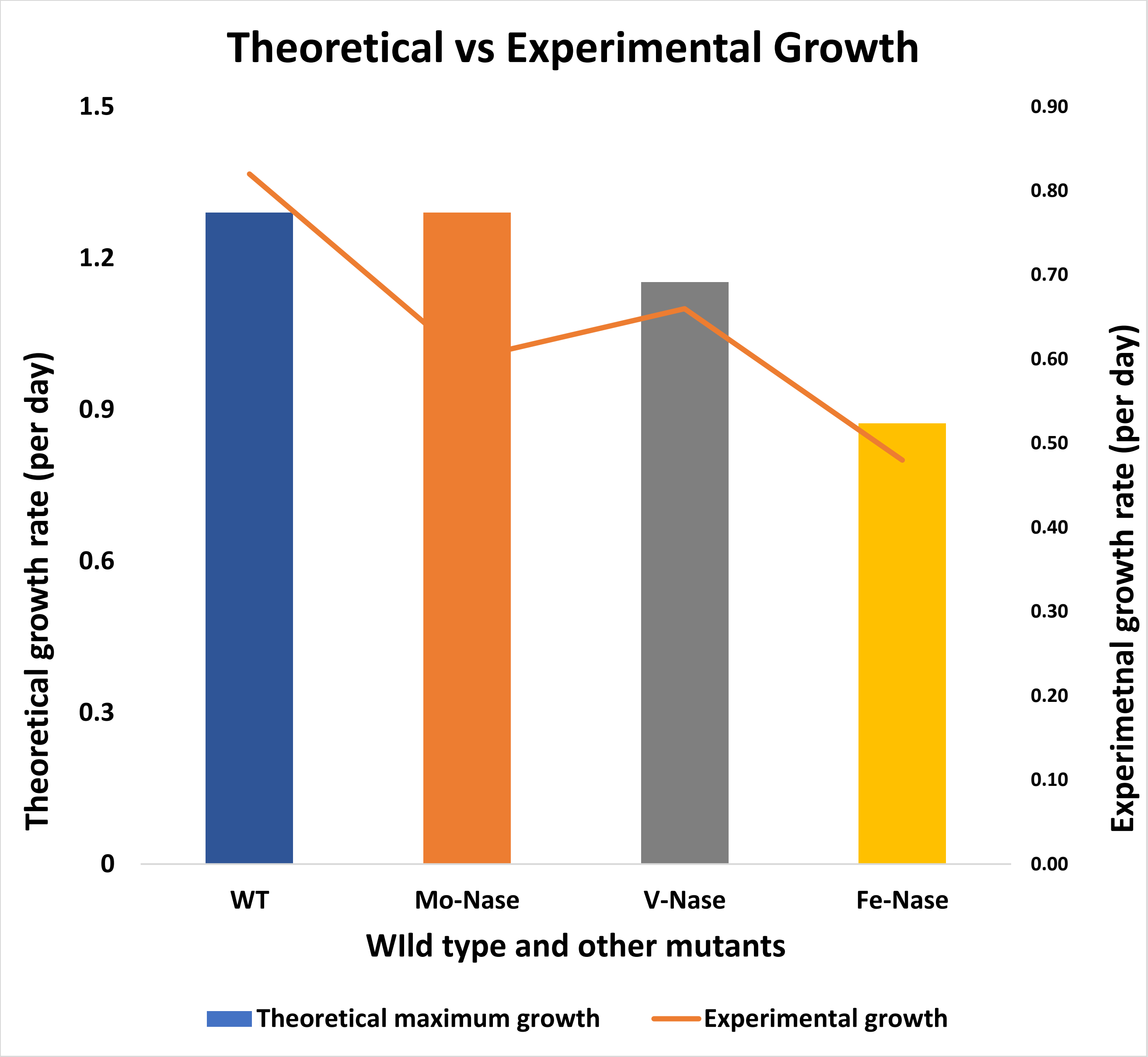

FIG S3 (e) For butyrate uptake, comparison between theoretical maximum growth found from the ME-modeling framework and the experimental work from Luxem et al. 2020 when acetate was used as substrate.

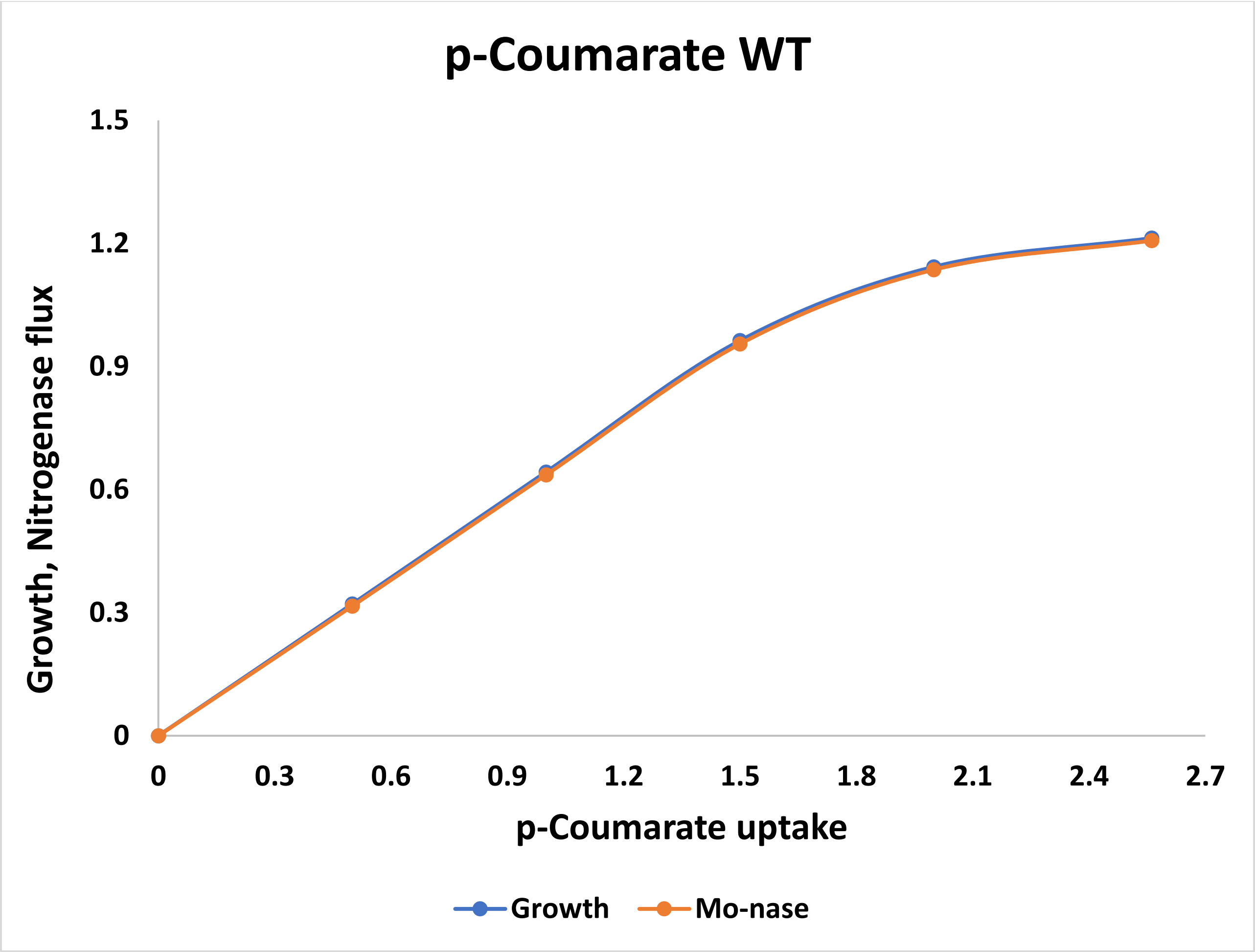

FIG S4 (a) For *p*-Coumarate uptake growth rate and nitrogen fixation rate for (a) WT *R. palustris*. For each of the case, growth rate and nitrogen fixation closely follow each other

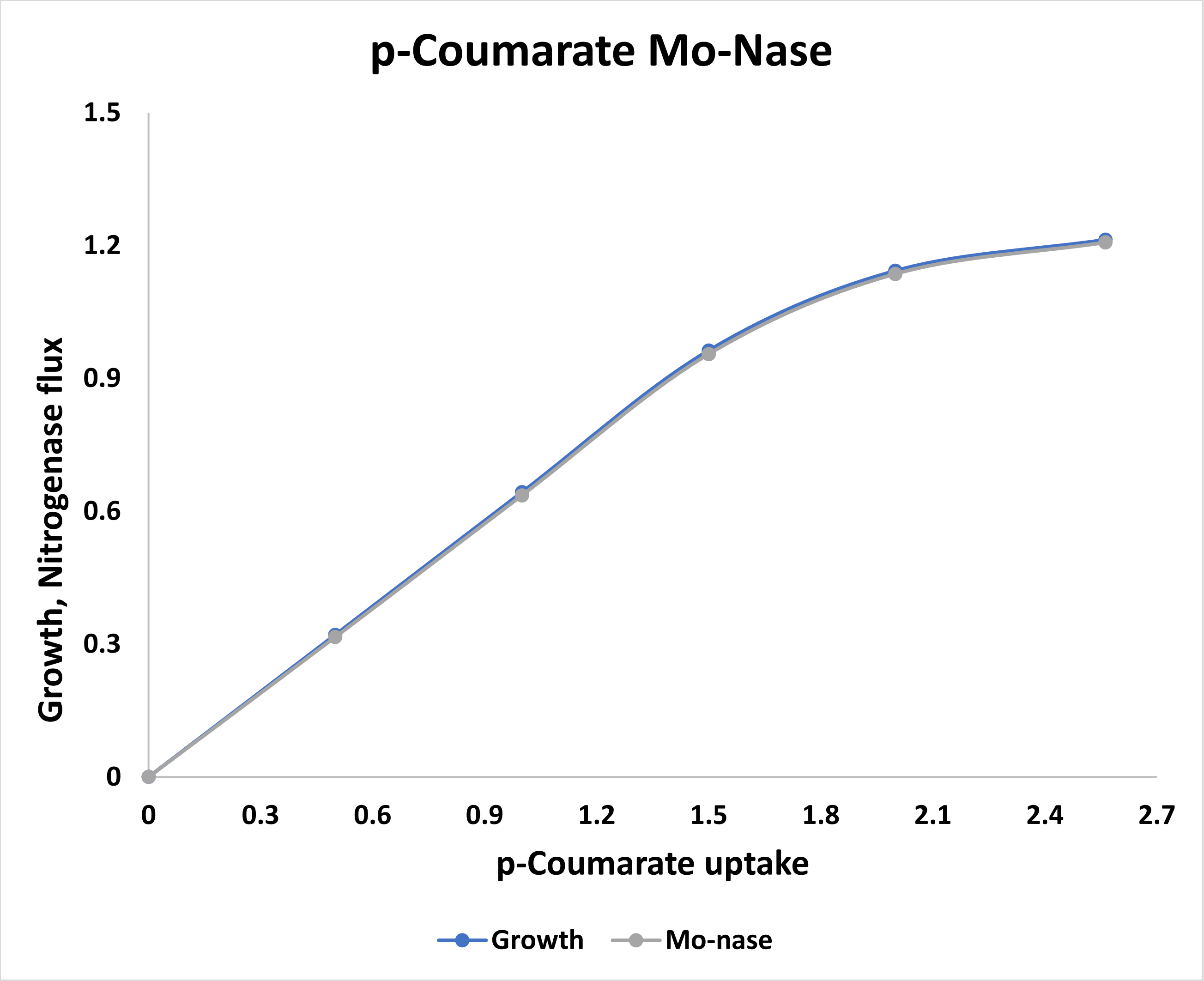

FIG S4 (b) For *p*-Coumarate uptake growth rate and nitrogen fixation rate for Mo-only mutant. For each of the case, growth rate and nitrogen fixation closely follow each other.

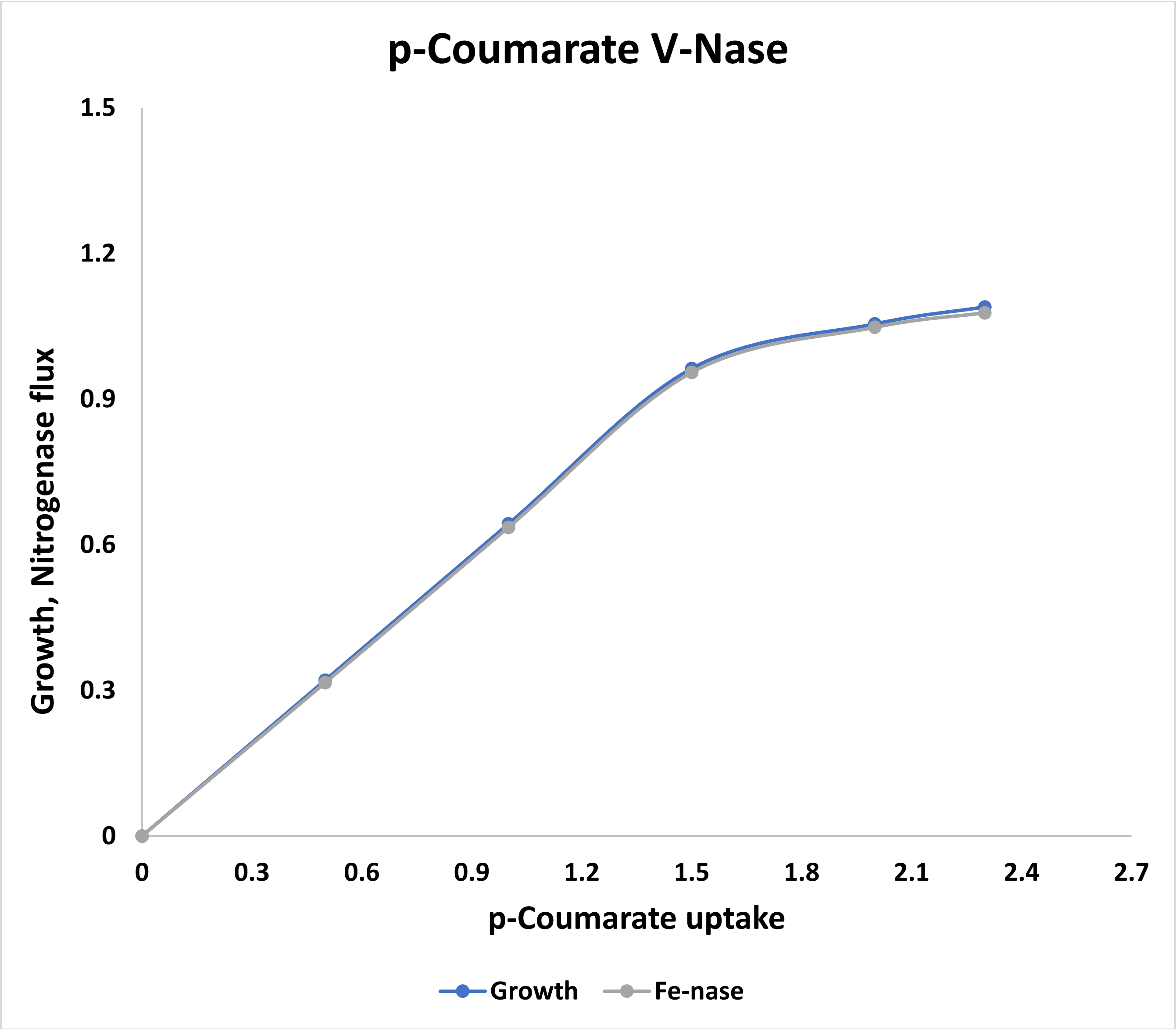

FIG S4 (c) For *p*-Coumarate uptake growth rate and nitrogen fixation rate for V-only mutant. For each of the case, growth rate and nitrogen fixation closely follow each other

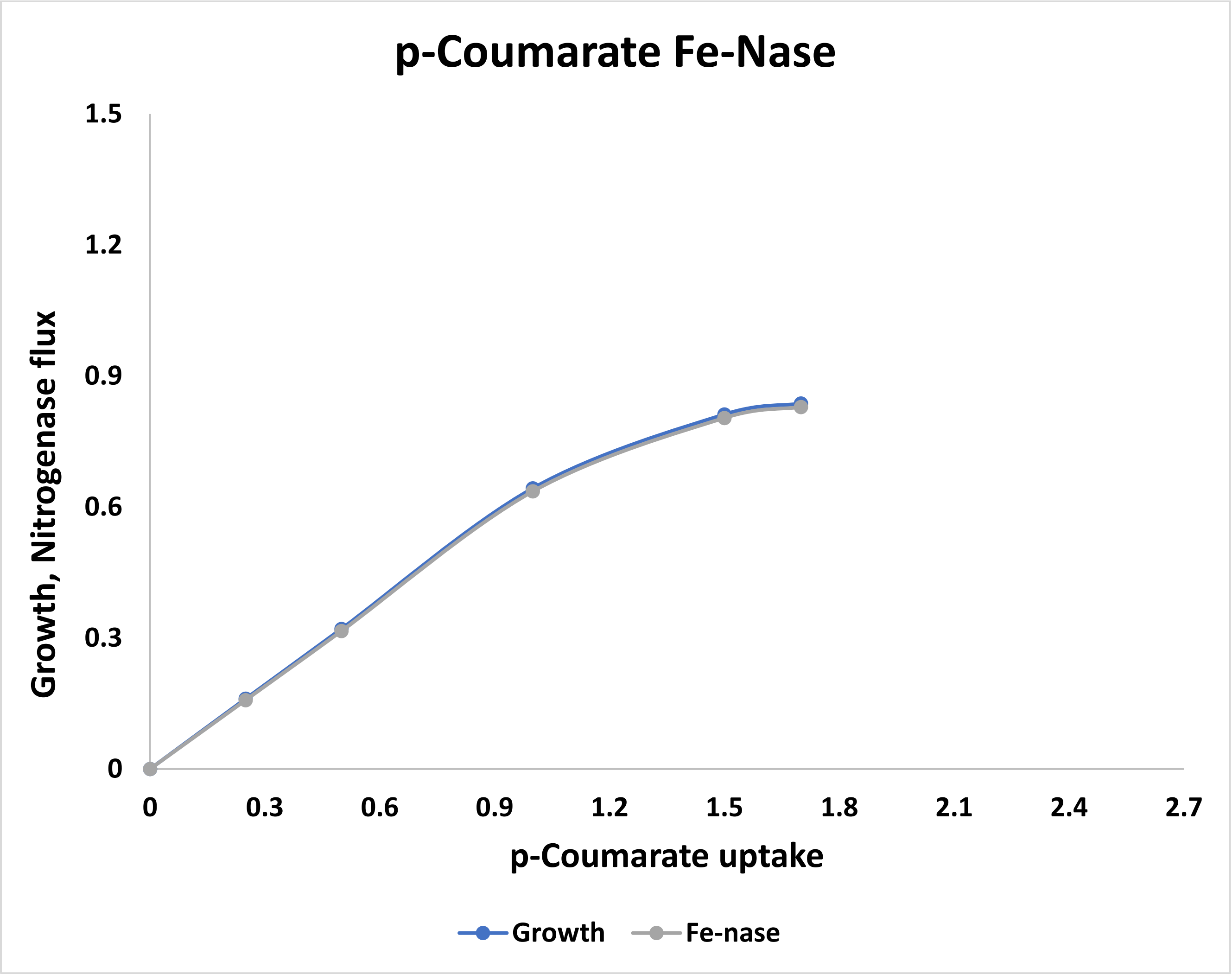

FIG S4 (d) For *p*-Coumarate uptake growth rate and nitrogen fixation rate for Fe-only mutant. For each of the case, growth rate and nitrogen fixation closely follow each other.

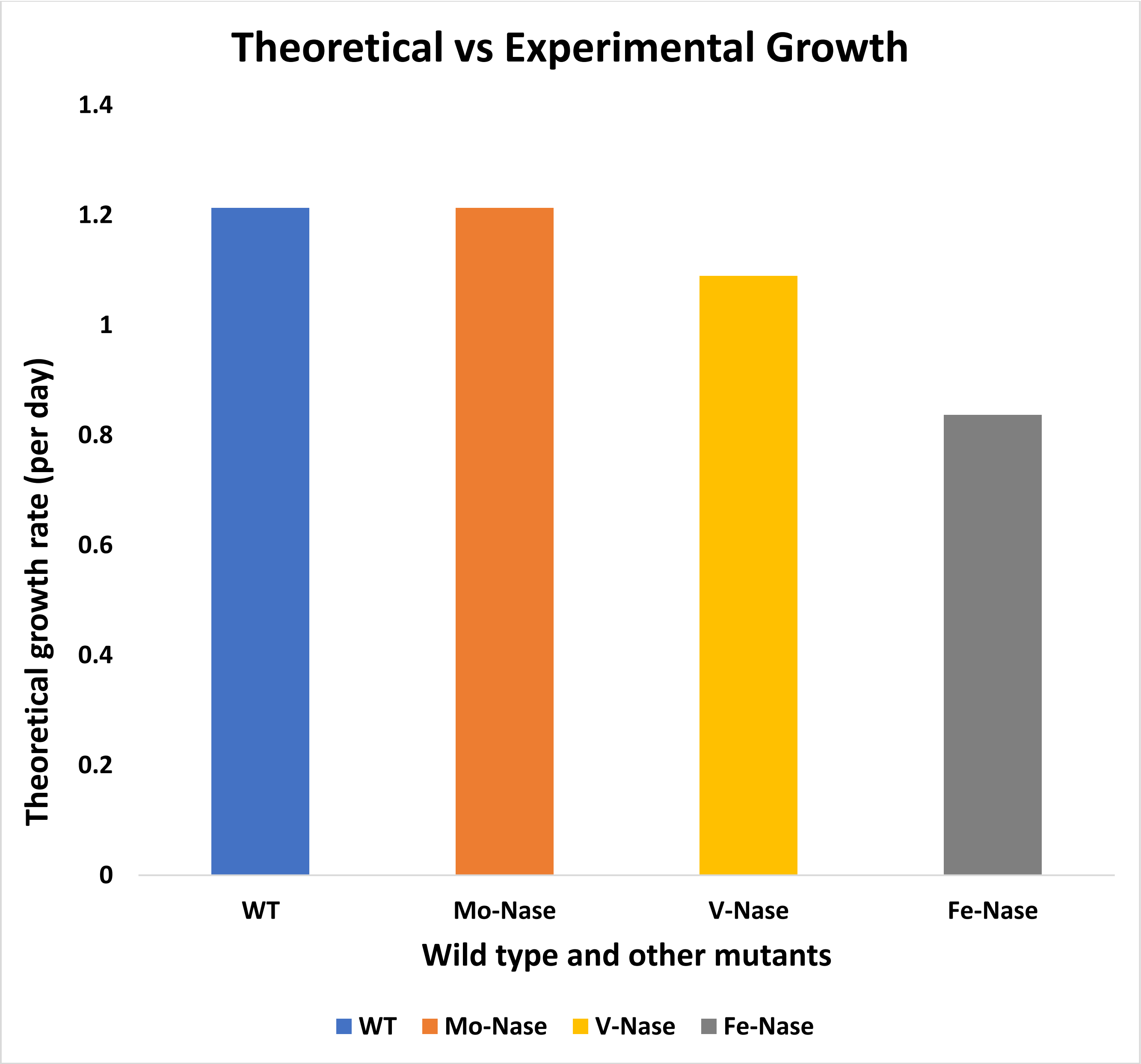

FIG S4 (e) For *p*-Coumarate uptake, theoretical maximum growth found from the ME-modeling framework for WT *R. palustris* and other -only mutants.
