# Supplementary material for: Characterizing the Interplay of Rubisco and Nitrogenase Enzymes in Anaerobic-Photoheterotrophically Grown Rhodopseudomonas palustris CGA009 through a Genome-Scale Metabolic and Expression Model
Source: Microbiol Spectr. 2022 Jun 22;10(4):e01463-22. doi: 10.1128/spectrum.01463-22 (PMC9431616; doi:10.1128/spectrum.01463-22)
Supplement: Supplemental file 6 — Supplemental material. Download spectrum.01463-22-s0005.pdf, PDF file, 0.1 MB [file spectrum.01463-22-s0005.pdf]

Transcription Reaction:

Coefficient of ribosome consumption = $\frac{l_{TU,i}}{3c_{ribo}K_T}(\mu + r_0 K_T)$

Here,

$l_{TU,i}$ = Number of nucleotides in each mRNA.

$c_{ribo} = \frac{m_{rr}}{m_{aa}f_{rRNA}}$, where m_{rr} is mass of rRNA per ribosome, m_{aa} is molecular weight of average amino acid, and $f_{rRNA}$is the fraction of RNA that is rRNA. The value of these parameters were chosen as following from the literature (1):

$m_{rr} = 1700\ KDa$, $m_{aa} = 109\ Da$, and $f_{rRNA} = 0.86$

μ = Growth rate.

r_0 = The value of this dimensionless constant was set to 4.5 (1).

K_T = This is defined as the effective catalytic constant which connects the RNA-to-Protein ratio with the growth rate. The value of this parameter was set to $4.5\ h^{-1}$ (1).

Translation Reaction:

Coefficient of mRNA consumption = $\frac{\mu + r_0 K_T}{3c_{mRNA}K_T}$

Here,

$c_{mRNA} = \frac{m_{nt}}{m_{aa}f_{mRNA}}$, where m_{nt} is the average molecular mass of RNA nucleotide, and f_{mRNA} is the fraction of RNA that is mRNA. The value of these parameters were chosen as following from the literature (1):

$m_{nt} = 324\ Da$, and $f_{rRNA} = 0.02$

Coefficient of amino acid consumption = $\frac{l_{p,i}}{c_{ribo}K_T}(\mu + r_0 K_T)$

Here,

$l_{p,i}$ = Number of amino acids in each proteins.

Metabolic Reaction:

Coefficient of mRNA consumption = $\frac{\mu}{k_{cat}}$

Here,

k_{cat} = Effective turnover rate. The mean effective turnover rate used in this study is $234000\ day^{-1}$. For different enzymes, this mean effective turnover rate was modified based on the molecular weight of the enzyme.

Reference:

1. O'Brien EJ, Lerman JA, Chang RL, Hyduke DR, Palsson BØ. Genome-scale models of metabolism and gene expression extend and refine growth phenotype prediction. Mol Syst Biol [Internet]. 2013 Jan 1;9(1):693. Available from: https://doi.org/10.1038/msb.2013.52

Metabolite pool size calculation:

Let's assume there are thee reactions as following"

Reaction 1: $C \rightarrow \ A$

Reaction 2: $D \rightarrow \ 3A$

Reaction 3: $A \rightarrow \ 4E$

Here, in reaction 1 and 2, A is being produced and in reaction 3, A is being consumed. If the flux of reaction 1 is $v_1 = 1 \ \frac{mmol}{gDW.day}$ and the flux of reaction 2 is $v_2 = 3 \ \frac{mmol}{gDW.day}$ then applying the pseudo steady state principles, the flux of reaction 3 will be 10 $\frac{mmol}{gDW.day}$. Then, the overall pool size of A will be $v_3 = 10 \ \frac{mmol}{gDW.day}$.

The idea is demonstrated in the following figure.

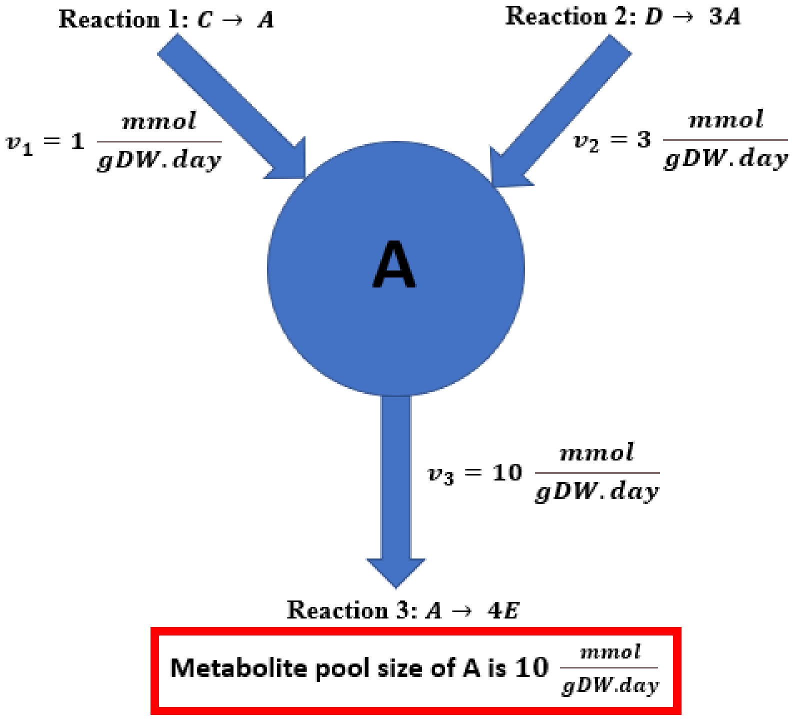

FIG S5 Demonstrating the calculation of metabolite pool size. Here A is a fictitious metabolite that is produced from two reactions, 1 and 2. Reaction 1 has a flux of 1 $\frac{mmol}{gDW.day}$ and reaction 2 has a flux of 3 $\frac{mmol}{gDW.day}$. A is only consumed in reaction 3. Applying pseudo steady mass balance assumption, flux of reaction 3 will be 10 $\frac{mmol}{gDW.day}$. Hence, the metabolite pool size of A is 10 $\frac{mmol}{gDW.day}$.
