# Supplementary material for: Characterizing the Interplay of Rubisco and Nitrogenase Enzymes in Anaerobic-Photoheterotrophically Grown Rhodopseudomonas palustris CGA009 through a Genome-Scale Metabolic and Expression Model
Source: Microbiol Spectr. 2022 Jun 22;10(4):e01463-22. doi: 10.1128/spectrum.01463-22 (PMC9431616; doi:10.1128/spectrum.01463-22)
Supplement: Supplemental file 7 — Supplemental material. Download spectrum.01463-22-s0006.pdf, PDF file, 0.3 MB [file spectrum.01463-22-s0006.pdf]

Simulation platform of the ME-model

Niaz Bahar Chowdhury [a], Adil Alsiyabi [a], and Rajib Saha [a] [#]

[a] Chemical and Biomolecular Engineering, University of Nebraska-Lincoln, Lincoln, Nebraska, 68508, USA.

[#]Address correspondence to Rajib Saha, rsaha2@unl.edu

Before You Begin

In this ME-model of *R. palustris*, the coefficients of metabolites in the metabolic reactions and coefficients of reactants in the translation and transcription reactions are not is the same order of magnitude. Thus, the flux of metabolic reactions and transcription/translation reactions are also not in the same order of magnitude. The highest ratio of metabolic reaction flux to transcription/translation reaction flux can be as high as in the order of 10^{17}, which is far higher compared to other ME-models (1,2). To solve this issue, the authors tried to use Qsopt_ex (3) to solve the ME-model, as it was used to solve the ME-model of *Thermotoga maritima* (1). However, Qsopt_ex is no longer compatible with the recent version of python. Note that, the publisher of Qsopt_ex suggested on their GitHub page to install a python module, Cython, before installing the Qsopt_ex. But installing Cython did not solve the issue and Qsopt_ex remained incompatible with the current version of Python. Even installing an older python version would not help solve the issue with Qsopt_ex. qMINOS (4) could not solve the problem to optimality either. From these observations, the authors strongly believe that an alternate platform is required to solve a ME-model where the ratio between metabolic reaction flux to transcription/translation reaction flux can be as high as in the order of 10^{17}.

GAMS is a very popular platform among the process systems engineering research community for solving large-scale optimization problems and GAMS files can be simulated without having to buy any license using GAMS Studio and NEOS Server. The authors used GAMS platform along with CPLEX solver to see if that can solve the ME-model to global optimality. In the preprocessing step of solving the optimization problem, CPLEX simplifies constraints, reduces problem size, and eliminates redundancy through numerical scaling. CPLEX's pre-solver attempts to reduce the size of a problem by inferring the nature of an optimal solution to the problem. Its aggregator uses substitution to try to eliminate variables and rows. Preprocessing is beneficial to total solution speed for most models, solving an optimization problem with scaling issues, and CPLEX reports the model's solution in terms of the user's original formulation, making the exact nature of any reductions irrelevant. When the model was solved in GAMS with CPLEX, the authors faced no such numerical issues, and the model was solved to the global optimality.

Thus, not only to repeat the growth rate results mentioned in Table 1 of the manuscript but also to use this model by the community, the preferred way will be to use the GAMS Studio and the NEOS Server interface. **Solving GAMS files in NEOS Server using GAMS Studio do not require purchasing any license.** In addition, interested users can convert the GAMS files to python pyomo files too (python users will at least need to run the GAMS model once to generate the necessary input files). Below is the step-by-step procedure for running GAMS files (steps 1-10) and python files (steps 11-21):

STEP 1:

Download the GAMS according to the configuration of your computer from the GAMS website (https://www.gams.com/download/).

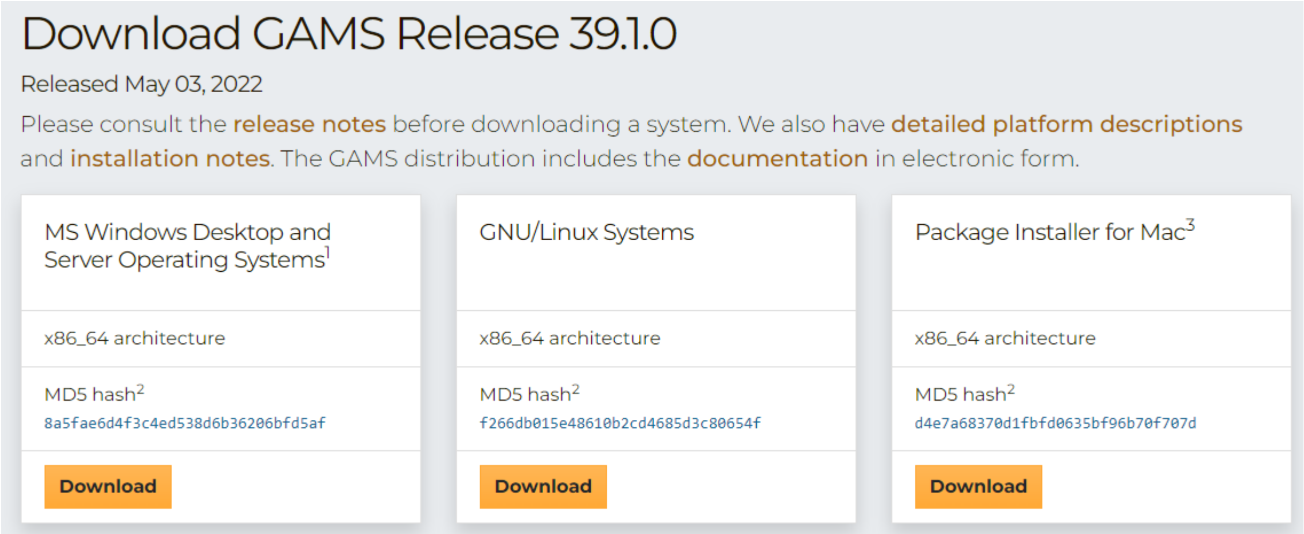

STEP 2:

Install the GAMS in your local hard drive. While installing, make sure to choose GAMS Studio.

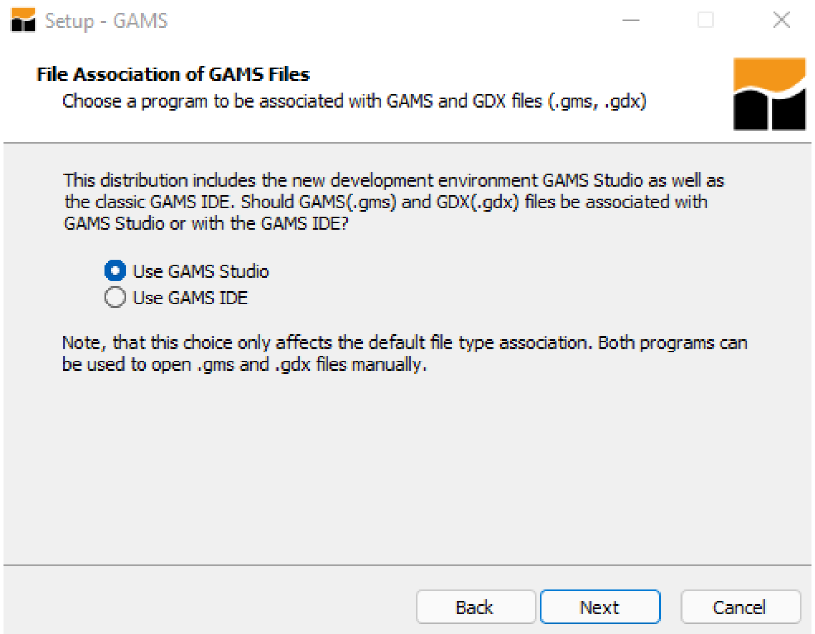

STEP 3:

Download all the relevant GAMS files from the GitHub directory (https://github.com/ssbio/palustris_ME_model/tree/main/GAMS).

ssbio Add files via upload

..	
cplex.opt	Add files via upload
lower_bound.txt	Add files via upload
metabolites.txt	Add files via upload
palustris_ME.gms	Add files via upload
reactions.txt	Add files via upload
sij.txt	Add files via upload
upper_bound.txt	Add files via upload

STEP 4:

The uptake rate of butyrate (line 3), succinate (line 6), p-coumarate (line 14), and acetate (line 48) can be set by changing the existing uptake rate. Different uptake rates and associated growth rates can be found in the supplementary file Table S3.

```
3   *BUTYRATE
4   'EX_cpd00211_e0_B'      0
5   *SUCCINATE
6   'EX_cpd00036_e0_B'      0
```

```
13   *p-COUMARATE
14   'EX_cpd00604_e0_B'      2
```

```
47   *ACETATE
48   'EX_cpd00029_e0_B'      0
```

STEP 5:

In the palustris_ME.gms file, In line 53, change the growth rate for different substrate uptake rates as mentioned in the supplementary file Table S3.

```
53   mu      Setting up the growth rate      /1.14/
```

STEP 6:

Click on the GAMS option and then click on the Run NEOS. This will automatically activate NEOS Server in the GAMS interface.

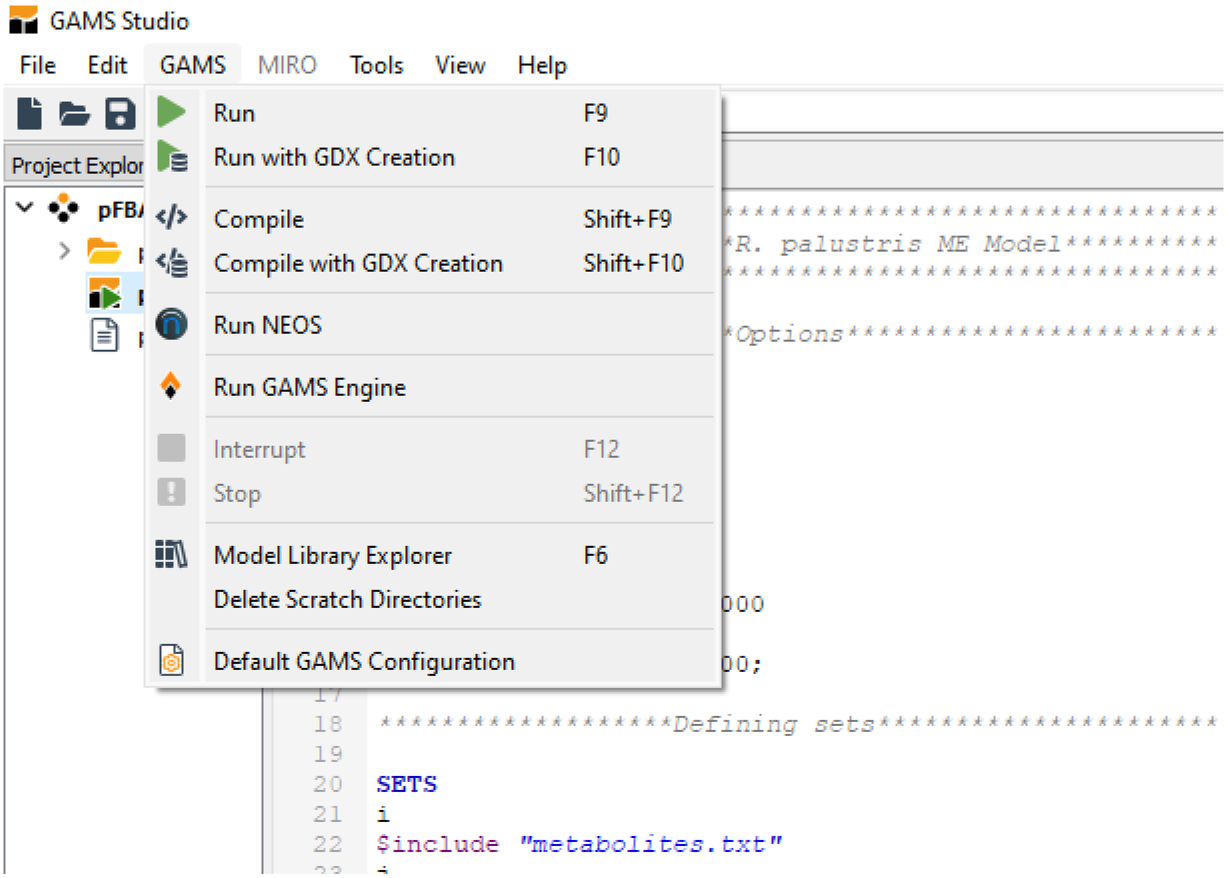

STEP 7:

Enter your email address in the pop-up window and then click on the Short priority. Short priority was chosen because the code will take less than 5 minutes to run.

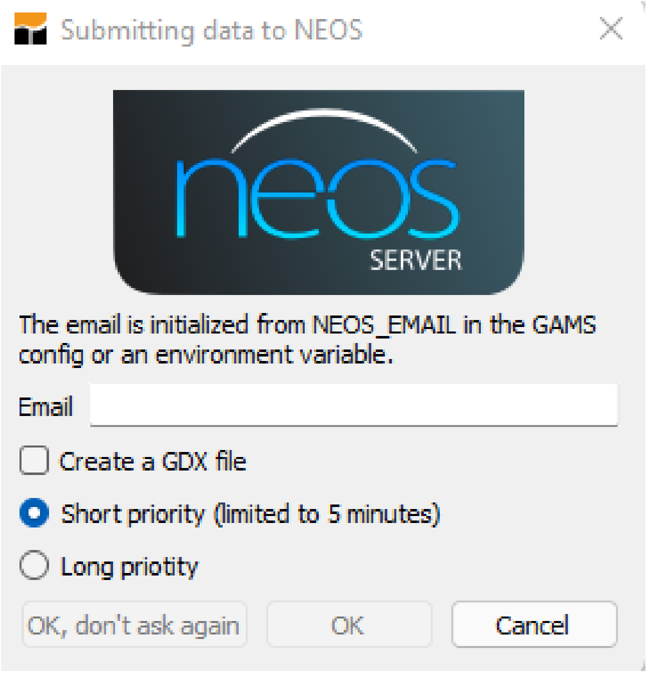

STEP 8:

Enter your email address and press okay. After that, the optimization problem will start and in the Process Log side window, the following message will show up that will indicate the optimality of the solution.

```
Solved to optimality.
--- Reading solution for model primal
--- Executing after solve: elapsed 0:00:01.118
*** Status: Normal completion
--- Job MODEL.gms Stop 05/07/22 20:22:48 elapsed 0:00:01.118
Composing results.
```

STEP 9:

With credentials communicated via e-mail from the NEOS Server, go to the bottom of the page and click on the Additional Output link and a compressed zip file should be downloaded.

```
**** FILE SUMMARY

Restart    /var/lib/condor/execute/dir_13923/gamsexec/restart.g00
Input      /var/lib/condor/execute/dir_13923/gamsexec/MODEL.gms
Output     /var/lib/condor/execute/dir_13923/gamsexec/solve.lst
```

Additional Output:
11932987-SkXEmVer-solver-output.zip

STEP 10:

From the compressed zip file, download the “r_palustris_ME_result.txt” file and all the flux distribution can be accessed from the “r_palustris_ME_result.txt” file.

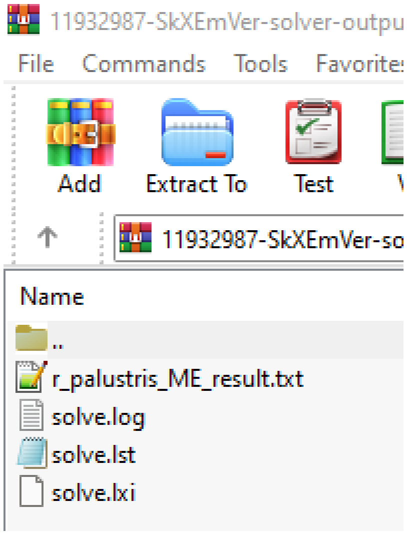


```
model status:        1.00
primal:1414.2994

CO2 pool:        6.51

nadh pool:       81.27

nadph pool:       79.41

atp pool:       85.40

pyr pool:       79.53

accoa pool:        7.22

pep pool:       78.34

mal pool:        7.15

oaa pool:        7.20

reaction fluxes
v('EX_cpd00211_e0_B')         0.00000000
v('EX_cpd00036_e0_B')         0.00000000
v('EX_cpd00528_e0_B')         1.44306088
v('EX_cpd00011_e0_B')         0.00000000
v('EX_cpd00013_e0_B')         0.00000000
v('EX_cpd00604_e0_B')         2.54000000
v('EX_cpd00067_e0_B')         0.00000000
v('EX_cpd00001_e0_B')         4.47657420
```

A copy of these files will also be automatically downloaded in the directory from which GAMS files were run.

The authors also recognize that some interested readers may want to run the ME-model in python format. This can be achieved as follows:

STEP 11: Open the text file "upper_bound.txt" from the folder (https://github.com/ssbio/palustris_ME_model/tree/main/GAMS). The uptake rate of butyrate (line 3), succinate (line 6), p-coumarate (line 14), and acetate (line 48) can be set by changing the existing uptake rate. Different uptake rates and associated growth rates can be found in the supplementary file Table S3.

```
3   *BUTYRATE
4   'EX_cpd00211_e0_B'      0
5   *SUCCINATE
6   'EX_cpd00036_e0_B'      0

13  *p-COUMARATE
14  'EX_cpd00604_e0_B'      2

47  *ACETATE
48  'EX_cpd00029_e0_B'      0
```

STEP 12: From the GitHub page (https://github.com/ssbio/palustris_ME_model/tree/main/GAMS), open the file, palustris_ME.gms. This is a GAMS script in which information on reactions, and metabolites were incorporated to simulate the ME-model. In line 53, change the growth rate for different substrate uptake rates as mentioned in the supplementary file Table S3.

```
53   mu      Setting up the growth rate      /1.14/
```

STEP 13: Just after line 8709, add the following: "option LP=convert;"

```
8709   primal.optfile=1;
```

STEP 14: Create a file named "convert.opt". Inside the file, add the following line "pyomo primal.py"

STEP 15: Follow STEPS 1-10, as shown earlier on how to connect GAMS Studio and NEOS server.

STEP 16: Once the GAMS file, "palustris_ME.gms", is simulated in the NEOS server environment, it will provide a Python Pyomo format file corresponding to the assigned growth rate and associated nutrient uptake rate.

STEP 17: Download or install the pyomo module in python. If you are a windows user, type the following command to install the pyomo module python.

“-m pip install pyomo”

STEP 18: As the ME-model is a large-scale optimization model, it can exceed the default maximum recursion depth of python. To make sure, it does not exceed the maximum recursion depth, in the load_model.py, import the sys module and add the following line. sys.setrecursionlimit(10**4).

```
#!/bin/env python

import sys
sys.setrecursionlimit(10**4)
```

STEP 19: The growth rate can be found in line 2431 of the palustris_ME.py file. Also, the substrate and essential nutrients are listed between lines 25 to 48 of the palustris_ME.py file. A dictionary (dictionary.txt) is also attached to identify each of the metabolites and reactions of the model.

```
2431   m.x2408 = Var(within=Reals,bounds=(1.14,1.14),initialize=1.14)
```

```
25  m.x2 = Var(within=Reals,bounds=(0,0),initialize=0)
26  m.x3 = Var(within=Reals,bounds=(0,0),initialize=0)
27  m.x4 = Var(within=Reals,bounds=(0,100),initialize=0)
28  m.x5 = Var(within=Reals,bounds=(0,0),initialize=0)
29  m.x6 = Var(within=Reals,bounds=(0,0),initialize=0)
30  m.x7 = Var(within=Reals,bounds=(0,2),initialize=0)
31  m.x8 = Var(within=Reals,bounds=(0,100),initialize=0)
32  m.x9 = Var(within=Reals,bounds=(0,100),initialize=0)
33  m.x10 = Var(within=Reals,bounds=(0,100),initialize=0)
34  m.x11 = Var(within=Reals,bounds=(0,100),initialize=0)
35  m.x12 = Var(within=Reals,bounds=(0,100),initialize=0)
36  m.x13 = Var(within=Reals,bounds=(0,100),initialize=0)
37  m.x14 = Var(within=Reals,bounds=(0,100),initialize=0)
38  m.x15 = Var(within=Reals,bounds=(0,100),initialize=0)
39  m.x16 = Var(within=Reals,bounds=(0,100),initialize=0)
40  m.x17 = Var(within=Reals,bounds=(0,100),initialize=0)
41  m.x18 = Var(within=Reals,bounds=(0,100),initialize=0)
42  m.x19 = Var(within=Reals,bounds=(0,100),initialize=0)
43  m.x20 = Var(within=Reals,bounds=(0,100),initialize=0)
44  m.x21 = Var(within=Reals,bounds=(0,100),initialize=0)
45  m.x22 = Var(within=Reals,bounds=(0,0),initialize=0)
46  m.x23 = Var(within=Reals,bounds=(0,100),initialize=0)
47  m.x24 = Var(within=Reals,bounds=(0,0),initialize=0)
48  m.x25 = Var(within=Reals,bounds=(0,0),initialize=0)
```

STEP 20: To update the photosynthetic efficiency for a given substrate, change the right-hand side of constraint m.c3191 (line 20507 to 205626). In this specific example, p-coumarate was used as a substrate and the right-hand side of constraint m.c3191 was changed to 85.4 mmol/gDW/day. Details of photosynthetic efficiency calculations for different substrates can be found in Materials and Methods section of the manuscript.

```
20507  m.c3191 = Constraint(expr=   m.x172 + m.x585 + m.x586 + m.x884 + m.x995 + m.x1037 + m.x1247 + m.x1473 + m.x1898
20508                              + m.x1909 + m.x1910 + m.x1915 + m.x1927 + m.x1936 + m.x1937 + m.x1938 + m.x1944 + m.x1956
20509                              + m.x1957 + m.x1985 + m.x2057 + m.x2058 + m.x2107 + m.x2119 + m.x2129 + m.x2139 + m.x2143
20510                              + m.x2147 + m.x2159 + m.x2203 + m.x2219 + m.x2222 + m.x2235 + m.x2243 + m.x2272 + m.x2274
20511                              + m.x2313 + m.x2318 + m.x2371 + m.x2373 + m.x2531 + m.x3532 + m.x3533 + m.x3534 + m.x3535
20512                              + m.x3536 + m.x3545 + m.x3546 + m.x3553 + m.x3554 + m.x3581 + m.x3582 + m.x3606 + m.x3607
20513                              + m.x3610 + m.x3611 + m.x3647 + m.x3648 + m.x3684 + m.x3685 + m.x3686 + m.x3687 + m.x3688
20514                              + m.x3701 + m.x3702 + m.x3749 + m.x3750 + m.x3751 + m.x3752 + m.x3824 + m.x3825 + m.x3826
20515                              + m.x3947 + m.x3948 + m.x4004 + m.x4005 + m.x4006 + m.x4007 + m.x4008 + m.x4009 + m.x4010
20516                              + m.x4011 + m.x4012 + m.x4013 + m.x4014 + m.x4015 + m.x4016 + m.x4017 + m.x4018 + m.x4052
20517                              + m.x4053 + m.x4054 + m.x4094 + m.x4095 + m.x4096 + m.x4097 + m.x4098 + m.x4183 + m.x4184
20518                              + m.x4189 + m.x4190 + m.x4217 + m.x4218 + m.x4237 + m.x4238 + m.x4239 + m.x4240 + m.x4241
20519                              + m.x4251 + m.x4252 + m.x4257 + m.x4258 + m.x4313 + m.x4314 + m.x4323 + m.x4324 + m.x4325
20520                              + m.x4391 + m.x4392 + m.x4456 + m.x4457 + m.x4458 + m.x4464 + m.x4465 + m.x4466 + m.x4467
20521                              + m.x4468 + m.x4493 + m.x4494 + m.x4562 + m.x4563 + m.x4564 + m.x4565 + m.x4566 + m.x4654
20522                              + m.x4655 + m.x4656 + m.x4657 + m.x4658 + m.x4715 + m.x4716 + m.x4773 + m.x4774 + m.x4779
20523                              + m.x4780 + m.x4785 + m.x4786 + m.x4855 + m.x4856 + m.x4879 + m.x4880 + m.x4881 + m.x4882
20524                              + m.x4883 + m.x4890 + m.x4891 + m.x4908 + m.x4909 + m.x4915 + m.x4916 + m.x4923 + m.x4924
20525                              + m.x4945 + m.x4946 + m.x5007 + m.x5008 + m.x5112 + m.x5113 + m.x5114 + m.x5430 + m.x5431
20526                              + m.x5444 + m.x5445 == 85.4)
```

STEP 21: Once the pyomo file is executed, the result should be accessed from the terminal. Sample output is given below:

```
Problem:
- Name: unknown
  Lower bound: 1494.34059685817
  Upper bound: 1494.34059685817
  Number of objectives: 1
  Number of constraints: 3193
  Number of variables: 5480
  Number of nonzeros: 49786
  Sense: minimize
Solver:
- Status: ok
  Termination condition: optimal
  Statistics:
    Branch and bound:
      Number of bounded subproblems: 0
      Number of created subproblems: 0
  Error rc: 0
  Time: 1.3612802028656006
```

Thank you. If you have any questions or concerns regarding these steps, please reach out to us (rsaha2@unl.edu) with detailed explanations.

References:

1. Lerman JA, Hyduke DR, Latif H, Portnoy VA, Lewis NE, Orth JD, et al. In silico method for modelling metabolism and gene product expression at genome scale. Nat Commun [Internet]. 2012;3(1):929. Available from: https://doi.org/10.1038/ncomms1928

2. O'Brien EJ, Lerman JA, Chang RL, Hyduke DR, Palsson BØ. Genome-scale models of metabolism and gene expression extend and refine growth phenotype prediction. Mol Syst Biol [Internet]. 2013 Jan 1;9(1):693. Available from: https://doi.org/10.1038/msb.2013.52

3. Applegate DL, Cook W, Dash S, Espinoza DG. Exact solutions to linear programming problems. Oper Res Lett [Internet]. 2007;35(6):693–9. Available from: https://www.sciencedirect.com/science/article/pii/S0167637707000211

4. Ma D, Yang L, Fleming RMT, Thiele I, Palsson BO, Saunders MA. Reliable and efficient solution of genome-scale models of Metabolism and macromolecular Expression. Sci Rep [Internet]. 2017;7(1):40863. Available from: https://doi.org/10.1038/srep40863
